# Major components of RNAi gene families in *Oryza sativa* cultivar *Kitaake*: *In-silico* discovery and characterization

**DOI:** 10.1016/j.heliyon.2024.e40395

**Published:** 2024-11-14

**Authors:** Md Darun Naim, Md Alamin, Md Parvez Mosharof, Ahmed Imtiaj, Md Nurul Haque Mollah

**Affiliations:** a Bioinformatics Lab, Department of Statistics, Faculty of Science, University of Rajshahi, Rajshahi, 6205, Bangladesh; b Department of Botany, Faculty of Biological Sciences, University of Rajshahi, Rajshahi, 6205, Bangladesh

**Keywords:** Characterizations, Integrated bioinformatics approaches, *Kitaake* rice, Regulators, RNAi genes

## Abstract

**Background:**

The *Oryza sativa* cultivar *Kitaake* is a promising new model rice for research due to its short life cycle (9 weeks), adaptability to greenhouse conditions, readily accepts foreign genes, and its complete genome sequence is accessible, providing a valuable blueprint for researchers. However, its major RNA interference (RNAi) gene families (DCLs, AGOs, RDRs) have not yet been studied. These gene families influence target-specific protein-coding gene expression and biotic and abiotic stresses, regulating plant growth and development.

**Objectives:**

This study aims to identify and characterize RNAi gene families from the *Kitaake* rice.

**Methods:**

This study has been designed by *in-silico* analysis to explore major RNAi genes highlighting their molecular functions, phylogenetic groups, regulatory factors, and other vital characteristics of *Kitaake* rice corresponding to OsRNAi genes.

**Results:**

This study has identified 10 DCLs, 21 AGOs, and 7 RDRs as major RNAi proteins of *Kitaake* rice corresponding to OsRNAi by BLASTP search. Domain analysis has been revealed the RNase III, PAZ, and Piwi domains are related to gene silencing processes. According to synteny study, *Kitaake* and rice have the most homology in 33 RNAi gene pairs, suggesting they share a chromosomal order and similar functions. The majority of OsKRNAi proteins have been located in the nucleus and chloroplast, which are related to gene silencing. Gene silencing and ribonuclease III activity are key terms from gene ontology (GO) analysis, which is part of the gene silencing process. Gene regulatory analysis has identified some important transcription factors including ERE, which participates in DNA binding and microRNAs (miRNAs) including 'Osa-MIR168' improves rice resistance to blast disease. The investigation of *cis*-acting regulatory elements in OsKRNAi genes has shown various crucial components, including MBS, W-box, LTR, ABRE, ARE that are linked with the different stresses. Genes (such as *OsKAGO1d*) were found to be overexpressed in target of rapamycin (TOR), increasing susceptibility to pathogens.

**Conclusion:**

The findings of this study may be useful resources for further experimental investigation on the improvement of *Kitaake* rice crop against different stresses.

## Abbreviations

ABRE: ABA-responsive element
AGO: Argonaute
ARE: Anaerobic Response Element
BLASTP: Basic Local Alignment Search Tool for Protein
DCL: Dicer like
DNA: Deoxyribonucleic acid
ERE: Estrogen response element
ERF: Ethylene responsive factors
LTR: Low Temperature Response
MBS: MYB binding site
OsRNAi: *Oryza sativa* RNAi
RDR: RNA-dependent RNA polymerase
W-box: Deoxyribonucleic acid (DNA) *cis*-regulatory element sequence, (T)TGAC(C/T)

## 1 Introduction

Rice, a basic meal for more than half of the world's population, is farmed in over 100 nations, with Asia producing 90 % [1]. The *Kitaake* rice (*O. sativa* cv *Kitaake*), a model plant for rice research, is known for its rapid flowering (9 weeks from seed to seed) and ease of cultivation [2,3]. Wet-lab experiments using *Streptomyces*-treated infected rice seedlings showed an 88.3 % decrease in rice blast disease [4]. Climate forecasts suggest that 16 % of the rice-growing region will be affected by temperatures, which will increase to 27 % by 2050 [5]. According to reports, abiotic causes (flooding and drought) caused a 70 % decline yields in 2011 [6].

RNA interference (RNAi) is a common mechanism where various RNA molecules affect the expression of protein-coding genes, influencing plant development, growth, stress responses, and antimicrobial defense [7]. It can happen via three recognized pathways as micro-RNA (miRNA), endogenous small interfering RNA (siRNA), and PIWI-interacting RNA (piRNA) [8]. The three main RNAi molecules regarded as argonaute (AGO), RNA-dependent RNA polymerase (RDR), and Dicer-like (DCL) genes/proteins [9]. The RNAi mechanism is started when DCL cleaves a partially double-stranded stem-loop RNA, also referred to as double-stranded RNA (dsRNA), into 21–24 nt short RNA (sRNA) duplexes [10]. AGO is an important component of the RNA-induced silencing complex (RISC) [11] and RDR plays an important function in synthesizing dsRNAs from RNA templates [12]. There are different numbers of RNAi genes in the DCL, AGO, and RDR families in different species of plants. For example, there are 20 genes in *Arabidopsis* [13] and cucumber [14], 51 genes in Brassica species [15], 32 genes in rice [16], 19 genes in barley [17], 14 genes in date palm [18], 38 genes in foxtail millet [19], 22 genes in grapevine [20] and pepper [21], 25 genes in sweet orange [22], 36 genes in sugarcane [23], and 31 genes in tea [24] have been identified.

RNAi is essential for gene diversity, providing plants with adaptive characteristics by allowing them to react dynamically to biotic and abiotic factors. Plants can use the RNAi pathway to silence certain genes, whether endogenous or acquired from pathogens, allowing them to modify their gene expression swiftly. This adaptability allows plants to deal with various biotic problems, such as viral, bacterial, and fungal diseases, as well as abiotic pressures like salinity, drought, and temperature variations. Plants can increase their ability to survive and breed successfully in shifting environments by diversifying their gene regulatory mechanisms through RNAi [[25], [26], [27], [28], [29], [30], [31], [32]]. The sRNAs are synthesized by different OsDCLs in rice (*Oryza sativa*) [33]. *OsDCL3b* regulates short RNA synthesis, which is critical in regulating protein-coding gene expression. Rice is more susceptible to fungal pathogen infection when *OsDCL1a* is active [34,35]. For the formation of male gametophytes, rice *AGO18* is required [36]. In rice OsAGOs have been found to co-express with RNA processing genes such as DCL, double-stranded RNA binding (DRB), and RDR [37]. The miR2118, associated with *OsAGO1b*/*OsAGO1d*, is involved in rice development and reproduction [38]. The *OsAGO2* causes more susceptibility to infection by the rice black-streaked dwarf virus (RBSDV) in rice [39]. *OsAGO17* plays a role in rice reproductive development and could be influenced to increase yield [40]. The *OsRDR6* plays a role in defense against viral, bacterial, and fungal infections [41]. When the *Venturia inaequalis* (fungal pathogen) was treated with *VICE12*-dsRNA, conidia generation, colony size, and gene expression all decreased significantly [42]. RNAi-mediated transgenic tomato flower stalk fed to adult western flower thrips (pest) and is observed a higher mortality rate than those pests who consumed wild-type samples [43].

RNAi operates through the sequence-specific silencing of genes (viral coat protein genes, fungal *R-genes*, bacterial *hrpL* gene, and nematod's *16D10* effector gene), effectively turning off targeted genetic expressions by degrading complementary mRNA, has been effectively used to combat various biotic stresses, such as viruses (e.g., *Tomato Yellow Leaf Curl Virus*) [44], bacteria (e.g., *Pseudomonas syringae*) [45] fungi (e.g., *Fusarium oxysporum*) [46], and nematodes (e.g., *Meloidogyne* spp.) [47], resulting in improved crop resistance to biotic stresses. RNAi, which functions through the sequence-specific silencing of genes (*SOS1* gene, *DREB* gene, *HSFA2* gene, *NADPH* oxidase gene, and *HMA4* gene) and effectively turning off targeted genetic expressions by degrading complementary mRNA, has been used to combat various abiotic stresses, such as salinity [48], drought [49], high temperature [50], oxidative [51], and heavy metal stress [52], resulting in improved crop resistance to abiotic stresses.

The combined impacts of biotic and abiotic stresses cause significant damage to rice crops globally, with losses ranging from up to 50 % depending on the severity and type of stress [53]. To overcome this damage, *Kitaake* rice may require target-specific gene silencing of biotic and abiotic stressors, as discussed above for rice and other crops. This could increase *Kitaake* rice yield by improving resistance to both abiotic and biotic stresses. However, little is known about these major RNAi gene families in one of the most important commercial rice crops [54] As a result, the purpose of this study was to collect substantial data on the major RNAi gene families (DCL, AGO, and RDR) that may contribute to the growth and development of *Kitaake* rice.

## 2 Materials and methods

### 2.1 The data source and descriptions

To investigate OsKRNAi proteins (DCL, AGO, and RDR) from the *Kitaake* rice (*O. sativa* cv *Kitaake*) genome, this study has considered its genome/proteome sequences from the Phytozome genome database [55] with Phytozome genome ID: 499 (NCBI taxonomy ID: 39947 BioSample: PRJNA448171, weblink: https://phytozome-next.jgi.doe.gov/info/OsativaKitaake_v3_1). This dataset has produced by Jain et al. [56]. It has utilized in functional genomic studies for efficient, cost-effective identification of genes conferring specific phenotype [57]. This study has utilized this genome dataset to investigate OsKRNAi proteins by basic local alignment search algorithm for proteins (BLASTP) search with the query sequences of *O. sativa* RNAi (OsRNAi) proteins. A total of 32 OsRNAi genes/proteins (8 OsDCLs, 19 OsAGOs, and 5 OsRDRs) sequences have downloaded from the Rice Genome Annotation Project (RGAP) database (Rice Release 7) that is developed by Kawahara et al. [58].

### 2.2 Combined bioinformatics analyses

The integrated bioinformatics studies [22,59] BLASTP include search [60], multiple sequence alignment [61], phylogenetic tree modeling [62], functional domain analysis [63], the exon-intron composition of OsKRNAi genes [64], subcellular location, synteny analysis [65], gene ontology (GO) analysis [66], TFs analysis [67,68], CAREs analysis and miRNA analysis [69,70] have been performed to investigate major OsKRNAi genes (AGOs, DCLs and RDRs) and their characterization as discussed in detail in the following sections.

#### 2.2.1 *Identification* of OsKRNAi proteins

##### 2.2.1.1 Exploring OsKRNAi proteins by BLASTP search

Protein-protein BLAST (BLASTP) is a bioinformatics tool that has been used to find similar protein of interest. In this investigation, the *Kitaake* rice genome from the Phytozome genome database has been used [71]. Using query coverage (≥50), identity (≥50 %), coverage (≥50 %), and E-values (≥10E-10) the OsKRNAi protein sequences have been retrieved [22,72]. Query coverage and identity percentage thresholds have been used at a minimum of 50 %, to ensure high confidence in the sequence alignment. Additionally, an E-value cutoff of minimum 10E-10 was employed to filter out hits that are likely to occur by chance, thereby ensuring the significance of the results so that we use those criteria. The Phytozome genome database provided the *Kitaake* rice genome's genomic length, protein ID, CDS length, and encoded protein length. The predicted protein sequences' molecular weight, pI, and GRAVY have been identified using ExPASy [73].

##### 2.2.1.2 Phylogenetic tree construction of OsKRNAi proteins

A phylogenetic tree displays the evolutionary links between different organisms. The Clustal-W program has been used to create multiple sequence alignments of the OsKRNAi protein sequences [74] through MEGAX [75] software. The Neighbor-joining (NJ) approach has been appreciated for its simplicity and speed, making it ideal for huge datasets. It creates trees by iteratively combining pairs of taxa based on their genetic distances, allowing it to produce correct trees quickly (Weighted Neighbor Joining: A Likelihood-Based Approach to Distance-Based Phylogeny Reconstruction). By applying the NJ method to phylogenetic tree analysis of the aligned sequences [76], 1000 bootstrap repetitions [77] have been utilized to confirm the evolutionary connection.

#### 2.2.2 Characterization of OsKRNAi proteins

##### 2.2.2.1 Conserved domains and motifs of OsKRNAi proteins

Domains and motifs are commonly used in bioinformatics to predict protein function and categorize proteins into families. NCBI conserved domain database (CDD) [78] database has been used to find for conserved domains in OsKRNAi, OsRNAi, maize (Zm) RNAi, and tomato (Sl) RNAi proteins [79,80]. CDD result has been displayed by the TBtools (a Toolkit for Biologists integrating various biological data-handling tools) [81]. Multiple Expectation Maximization for Motif Elicitation (MEME-Suite) [82], an online software for protein sequence analysis has been used to analyze the conserved motif of OsKRNAi, OsRNAi, maize (Zm) RNAi, and tomato (Sl) RNAi proteins [79,80]. The following parameters have been defined for this purpose: (i) The best motif numbers are >6 to <50; (ii) the highest number of motifs is 10.

##### 2.2.2.2 OsKRNAi genes structures

Gene structures depict the exon-intron patterns of a gene, in which exons are the protein building blocks and introns are intervening sections that are deleted before the final protein product is produced. The Gene Structure Display Server (GSDS 2.0) [83] has been considered to establish the gene structure of the OsKRNAi genes. The structure of OsKRNAi genes has been compared with the OsRNAi gene structure through the exon-intron composition.

##### 2.2.2.3 Sub-cellular localization of OsKRNAi proteins

Subcellular localization is the particular location of a protein within a plant cell. The nucleus, mitochondria, chloroplasts, and cytoplasm all serve various functions inside a cell. The legend values indicate the number of proteins that are closest to the query proteins (OsRNAi/OsKRNAi) in different sub-cellular locations. To estimate the subcellular location of the OsKRNAi proteins, a web-based integrative subcellular location predictor tool called WoLF PSORT (https://wolfpsort.hgc.jp/) has been investigated. The values represent the nearest neighbor proteins to each site's query proteins. The nearest neighbor value in WoLF PSORT represents the similarity between your query protein and a known protein with a determined subcellular location. The TBtools have been utilized to show those data [81].

##### 2.2.2.4 Synteny analysis of OsKRNAi genes

Synteny analysis examines how genes are organized on chromosomes in various plant species. When genes from different species appear in the same order on their respective chromosomes, they are considered syntenic. It demonstrates that these genes are derived from the same ancestor and may have identical functions. Gene replication events have been detected by MCScanX, and the synteny of *Kitaake* rice has been visualised by TBtools. The whole genome data of *O. sativa*, *O. sativa Kitaake*, *Sorghum bicolor*, and *Zea mays* have aligned and analyzed using the TBtools software's One Step MCScanX tool. One Step MCScanX is a streamlined version of the MCScanX toolkit, designed to simplify the process of detecting synteny and collinearity between genomes. It combines multiple steps of the original MCScanX pipeline into a single, user-friendly process [81]. The gene pairs divergence time of has been determined using the synonymous mutation rate of substitutions per synonymous (Ks) site each year, which is calculated as follows: T = Ks/2x (x = 1.5∗10^-8) [84].

#### 2.2.3 Functional enrichment analysis of OsKRNAi proteins

Gene Ontology (GO) analysis is a computational tool for understanding the biological roles of genes. It aids in the classification and understanding of genes involved in a variety of plant activities such as growth, development, metabolism, stress response, and others. An online tool integrated into PlantTFDB has been used to assess the GO study and verify that OsKRNAi proteins participate in words related to molecular functions (MFs) and biological processes (BPs) [68]. PlantTFDB is a comprehensive resource for studying plant transcription factors. The database now includes PlantRegMap, a portal for genomic TF repertoires, high-quality binding motifs, genome-wide regulatory interactions, and research tools. Covering 165 species, PlantTFDB 5.0 has over 320,000 transcription factors in 58 families. *Kitaake* is a variety of *Japonica* rice and as a result, this GO analysis has been performed using the genome of the *O. sativa* subsp. *Japonica*. In this case, enriched GO-terms are already annotated for OsRNAi proteins but predicted for OsKRNAi proteins. Using Benjamini-Hochberg corrections, Fisher's exact test has been employed to measure the p-values. This investigation considers a GO term with a p-value less than 0.05 statistically significant. We independently verified the results for both OsRNAi and OsKRNAi using DeepGOWeb [85].

#### 2.2.4 OsKRNAi gene regulatory network analysis

Together, TFs, *cis*-elements, and miRNAs form a complex regulatory network that controls gene expression in plants where *cis*-elements are short DNA sequences located near the genes they regulate, these elements are the sites where TFs attach to either start or stop gene transcription. miRNAs are tiny RNA molecules that bind to particular mRNA sequences to cause translational inhibition or mRNA destruction. In this study, the associated TFs family with the OsKRNAi genes has been analyzed using Plant Transcription Factor Database (plantTFDB v4.0) (http://planttfdb.gao-lab.org/) [68,86]. Utilizing Cytoscape 3.9.0 for analysis, the regulatory network and sub-network have been generated by combining TFs and OsKRNAi genes [87]. Based on the degree of connectedness, this study has determined the hub genes and associated key hub TFs from the network. Plant CAREs database has conducted analysis on *cis*-element analysis [88]. Utilizing Plant miRNA ENcyclopedia (PmiREN), *Kitaake* rice mature_miRNA_expression and mature_miRNA_sequence have been downloaded [89]. Plant sRNA target (psRNATarget) [90] has been used in the analysis to determine the mature_miRNA_expression and mature_miRNA_sequence IDs that matched OsKRNAi genes. This data has been shown using the TBtools software [81].

#### 2.2.5 OsKRNAi gene expression analysis

*In silico* gene expression analysis is a computational method used to study the activity of genes within a plant without conducting physical experiments. It involves analyzing large datasets of genetic information, such as gene sequences and expression patterns, using specialized software and algorithms. The dataset for *Kitaake* rice can be accessed through the NCBI GEO database, specifically under the dataset (GSE93872) with the BioProject ID PRJNA362628 [91]. The TOR-specific inhibitor rapamycin was utilized to analyze the transcriptome in various locus expressions of *Kitaake* rice cells. This study is based on the NCBI-GEO dataset's OsKRNAi locus ID expression in *Kitaake* rice. Arbitrary fold change (FC) cutoffs of >2 have restricted data collection to genes that exhibit extreme variation from other genes. Log2 ratios, commonly known as Log2-fold-change (Log2FC) values, represent the expression data. The most frequently used tools for RNA-seq or microarray gene expression analysis make it simple to acquire log2FC values [92,93]. To identify differentially expressed genes in expression studies, thousands of genes in a genome-wide data collection are evaluated against a null hypothesis. The False Discovery Rate (FDR), which refers to the anticipated proportion of false positive genes within a given set of genes, has been proposed as a method for evaluating the statistical significance of such set [94]. The statistical significance of this dataset has been analyzed using FDR values of OsKRNAi expression data. This study has considered significant for *p*-values of <0.05 according to the two-tailed student's *t*-test.

## 3 Results

### 3.1 Identification of OsKRNAi proteins

In this study, a total of 10 OsKDCLs, 21 OsKAGOs, and 7 OsKRDRs have been identified as RNAi proteins from the genome of *Kitaake* rice. The OsRNAi proteins have been used as the query sequence in a BLASTp search, employing multiple sequence alignment (MSA) analysis and OsKRNAi proteins have been named by OsRNAi proteins (Table 1). Therefore, it has been considered these 38 OsKRNAi proteins for further investigation. For convenience of presentation, the *Kitaake* rice RNAi protein families have labeled as OsKDCLs, OsKAGOs, and OsKRDRs, while the *O. sativa* RNAi proteins were labeled as OsDCLs, OsAGOs, and OsRDRs. The names of 38 OsKRNAi proteins have been denoted as 10 OsKDCLs proteins (OsKDCL1, OsKDCL2, OsKDCL3a, OsKSHO1a, OsKSHO1b, OsKDCL3b1, OsKDCL3b2, OsKDCL3b3, OsKDCL3b4, OsKDCL3b5), 21 OsKAGOs proteins (OsKAGO1a1, OsKAGO1a2, OsKAGO1b1, OsKAGO1b2, OsKAGO1c, OsKAGO1d, OsKAGO2, OsKAGO3, OsKAGO14, OsKAGO4a, OsKAGO4b, OsKMEL1a, OsKMEL1b, OsKSHL4, OsKPNH1, OsKAGO12, OsKAG13, OsKAGO15, OsKAGO16, OsKAGO17, OsKAGO18) and 6 OsKRDRs proteins (OsKRDR1a, OsKRDR1b, OsKRDR2, OsKRDR3, OsKRDR4a, OsKRDR4b, OsKSHL2). The Phytozome database provides information on the lengths of OsKDCL genes, which vary from CDS 4137 (*OsKDCL3b2*) to 5778 (*OsKDCL1*) base pairs (bp) and the protein lengths of these genes range from 1378 to 1925 amino acids. The lengths of OsKAGO genes vary from CDS 1995 (*OsKAGO12*) to 3579 (*OsKAGO13*) base pairs, while their protein lengths range from 664 to 1192 amino acids. The OsKRDR genes have a range of lengths, varying from CDS 2223 (*OsKRDR4a*) to 3657 (*OsKSHL2*) base pairs (bp) and their protein lengths range from 740 to 1218 amino acids, as shown in Table 1. According to the ExPASy database, the OsKDCL proteins are acidic (since pI < 7.0) except OsKSHO1b*,* which is alkaline (since, pI > 7.0). All of the OsKAGO proteins are alkaline (pI > 7.0). The OsKRDR protein's pI values have shown that they are acidic except for OsKRDR3 and OsKRDR2 (pI > 7.0), which are alkaline (Table 1).

**Table 1:** Basic information of OsKDCL, OsKAGO and OsKRDR protein families of *Kitaake* rice. The protein names, transcript ID, chromosomal location, CDS length, and protein length (aa) were collected from the Phytozome database. The molecular weight, isoelectric point (pI), and grand average of hydropathicity (GRAVY) values were collected from the ExPASy database. Molecular weights are in Daltons (D), and "aa" means amino acid.

Serial number	Protein name	Transcript ID	Chromosomal location	CDS (bp)	No of Ex./Int.	Protein			
						Len (aa)	Molecular Wt. (D)	pI	GRAVY
**OsKAGO**									
1	OsKAGO1a1	OsKitaake02g283000.1	chr2:28066720-28078774	3249	23:22	1082	120448.7	9.46	−0.511
2	OsKAGO1a2	OsKitaake02g283000.2	chr2:28066720-28075544	3201	23:22	1066	118637.7	9.49	−0.516
3	OsKAGO1b1	OsKitaake04g228300.1	chr4:29721566-29736817	3357	22:21	1118	123592	9.55	−0.54
4	OsKAGO1b2	OsKitaake04g228300.2	chr4:29721566-29731350	3306	3:02	1101	121632.7	9.55	−0.538
5	OsKAGO1c	OsKitaake02g396600.1	chr2:36985221-36992610	3036	3:02	1011	113144.2	9.96	−0.458
6	OsKAGO1d	OsKitaake06g292000.1	chr6:30862968-30870424	3117	22:21	1038	115923.7	9.19	−0.452
7	OsKAGO2	OsKitaake04g260800.1	chr4:32537900-32542300	3105	3:02	1034	111444.7	9.38	−0.446
8	OsKAGO3	OsKitaake04g260900.1	chr4:32542902-32548439	3330	22:21	1109	120553.2	8.84	−0.517
9	OsKAGO14	OsKitaake07g057500.1	chr7:4637186-4646828	3150	22:21	1049	113525.5	9.64	−0.352
10	OsKAGO4a	OsKitaake01g124000.1	chr1:9789853-9797456	2715	22:21	904	100639.1	9.12	−0.395
11	OsKAGO4b	OsKitaake04g019500.1	chr4:4010389-4017951	2736	22:21	911	101740.2	9.07	−0.412
12	OsKMEL1a	OsKitaake03g377400.1	chr3:34674159-34682745	2541	20:19	846	96210.97	9.08	−0.428
13	OsKMEL1b	OsKitaake03g377400.2	chr3:34674159-34680729	2184	22:21	727	82484.34	9.02	−0.391
14	OsKSHL4	OsKitaake03g229100.1	chr3:19661486-19666201	3147	19:18	1048	117498.4	9.41	−0.38
15	OsKPNH1	OsKitaake06g203700.1	chr6:23140879-23154384	2946	22:21	981	108575.5	9.34	−0.369
16	OsKAGO12	OsKitaake03g292100.1	chr3:28246662-28252238	1995	9:08	664	75434.67	8.97	−0.381
17	OsKAG13	OsKitaake03g368700.1	chr3:34114215-34123400	3579	22:21	1192	135726.9	9.38	−0.312
18	OsKAGO15	OsKitaake01g123900.1	chr1:9772209-9786787	2628	21:20	875	97870.9	9.09	−0.429
19	OsKAGO16	OsKitaake07g096200.1	chr7:9388859-9402278	2664	21:20	887	99209.31	9.12	−0.288
20	OsKAGO17	OsKitaake02g055200.1	chr2:3822342-3829484	2631	23:22	876	98911.96	8.92	−0.235
21	OsKAGO18	OsKitaake07g137400.1	chr7:16738943-16745902	3267	21:20	1088	117940.6	9.33	−0.537
**OsKDCL**									
1	OsKDCL1	OsKitaake03g019000.1	chr3:1227716-1237412	5778	19:18	1925	215205.2	6.24	−0.384
2	OsKDCL2	OsKitaake03g241635.1	chr3:22167336-22182013	3798	19:18	1265	142776.9	6.57	−0.188
3	OsKDCL3a	OsKitaake01g441800.1	chr1:40636046-40646740	4956	24:23	1651	184892.4	6.4	−0.221
4	OsKDCL3b1	OsKitaake10g137500.3	chr10:19107443–19120053	5088	26:25	1695	190727.4	6.54	−0.167
5	OsKDCL3b2	OsKitaake10g137500.2	chr10:19107442–19120054	5082	26:25	1693	190471.1	6.54	−0.169
6	OsKDCL3b3	OsKitaake10g137500.1	chr10:19107443–19120053	5091	26:25	1696	190855.5	6.54	−0.169
7	OsKDCL3b4	OsKitaake10g137500.5	chr10:19109308–19120054	4143	20:19	1380	156022.7	6.33	−0.162
8	OsKDCL3b5	OsKitaake10g137500.4	chr10:19109308–19120054	4137	20:19	1378	155766.4	6.33	−0.157
9	OsKSHO1a	OsKitaake04g193100.2	chr4:26727003-26741042	4227	23:22	1508	170766.5	6.87	−0.222
10	OsKSHO1b	OsKitaake04g193100.1	chr4:26727003-26741042	4629	23:22	1542	175130.7	7.96	−0.213
**OsKRDR**									
1	OsKRDR1b	OsKitaake02g325100.2	chr2:5403157-5415516	2700	3:02	899	84255.38	6.6	−0.328
2	OsKRDR1a	OsKitaake02g325100.1	chr2:5398300-5415516	3552	3:02	1183	84255.38	6.6	−0.328
3	OsKRDR2	OsKitaake04g159700.1	chr4:24496070-24501967	3411	4:03	1136	126925.12	7.33	−0.254
4	OsKRDR3	OsKitaake01g069700.1	chr1:5379025-5391518	2472	15:14	823	92859.7	7.27	−0.293
5	OsKRDR4a	OsKitaake01g069900.2	chr1:31888099-31893083	2223	16:15	740	99531.65	6.12	−0.297
6	OsKRDR4b	OsKitaake01g069900.1	chr1:31885487-31893083	2223	20:19	740	132399.26	6.65	−0.363
7	OsKSHL2	OsKitaake01g192900.1	chr1:19292772-19298044	3657	2:01	1218	136534.17	6.69	−0.234

### 3.2 Characterization of OsKRNAi proteins

#### 3.2.1 Conserved domains and motifs analysis of OsKRNAi proteins

Protein domains are specific functional and structural subunits. This study has investigated the similarity between the conserved domains of the major OsKRNAi proteins and the OsRNAi proteins with the maize (Zm) RNAi and tomato (Sl) RNAi. Fig. 2, has been shown that all OsKDCLs, OsDCLs, ZmDCLs, and SlDCLs display almost similar conserved domains, including Helicase_C (Helicase conserved C-terminal domain), Dicer_dimer (Dicer dimerization domain), PAZ (Piwi/Argonaute/Zwille) domain, Ribonuclease 3 (hydrolyses RNA), ResIII (Restriction endonuclease III) domain, DND1-DSRM (double-stranded RNA binding motif of dead end protein homolog 1), DEAD (ATP-dependent RNA unwinding domain), dsrm (double-stranded RNA binding motif). It has been found that the OsKAGOs, OsAGOs, ZmAGOs and SlAGOs all have almost identical conserved domains, such as PAZ (Piwi/Argonaute/Zwille) domain, ArgoN (N-terminal domain of argonaute), ArgoL1 (Argonaute, linker 1), ArgoL2 (Argonaute, linker 2), ArgoMid (Mid domain of argonaute), PIWI (P-element induced wimpy testis), and Gly-rich_Ago1 (Glycine-rich region of argonaut) domains. It has been identified that the OsKRDRs, OsRDRs, ZmRDRs and SlRDRs all have displayed almost the same conserved domains, including the RdRP (RNA-directed RNA polymerase) domain (Fig. 2).

**Fig. 1:**
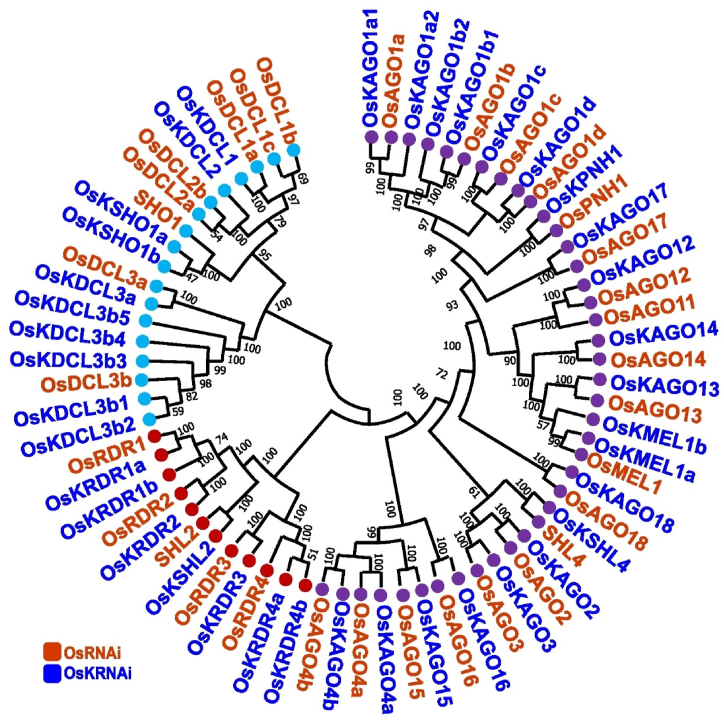
The combined phylogenetic tree. In this tree, the OsKRNAi (OsKDCLs, OsKAGOs, OsKRDRs) and OsRNAi (OsDCLs, OsAGOs, OsRDRs) proteins have been represented by blue and orange, respectively. Here DCLs, AGOs, and RDRs families have been represented by peste, purple and red circles, respectively.

**Fig. 2:**
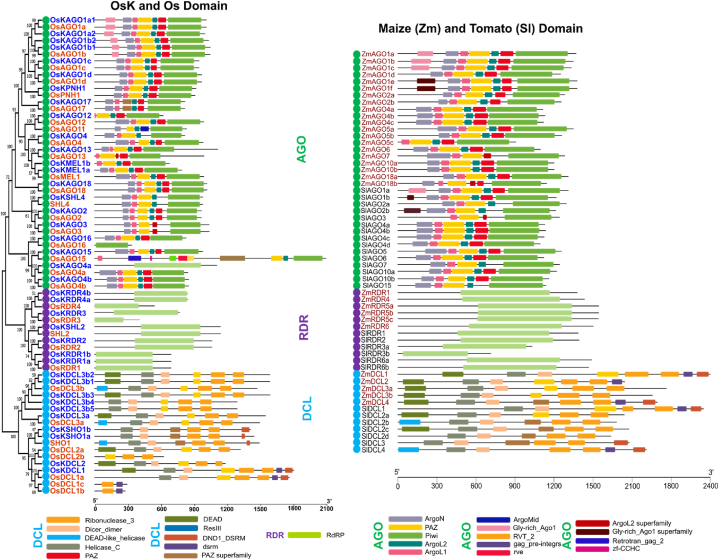
The conserved domains of OsRNAi, OsKRNAi, ZmRNAi, and SlRNAi proteins. Different colors represent different conserved domains. Note: Helicase_C - Helicase conserved C-terminal domain, Dicer_dimer - Dicer dimerization domain, PAZ - PAZ domain, RNase III - Ribonuclease III domain, DND1-DSRM - double-strand RNA binding domain from DEAD END PROTEIN 1, DEAD - DEAD/DEAH box helicase domain, dsrm - Double-stranded RNA binding motif, ArgoN - N-terminal domain of argonaute, ArgoL1 - Argonaute linker 1 domain, ArgoL2 - Argonaute linker 2 domain, ArgoMid - Mid domain of argonaute, PIWI - PIWI domain, Gly-rich_Ago1 - Glycine-rich region of Argonaut, RdRP - RNA dependent RNA polymerase, ResIII -Type III restriction enzyme domain.

**Fig. 3:**
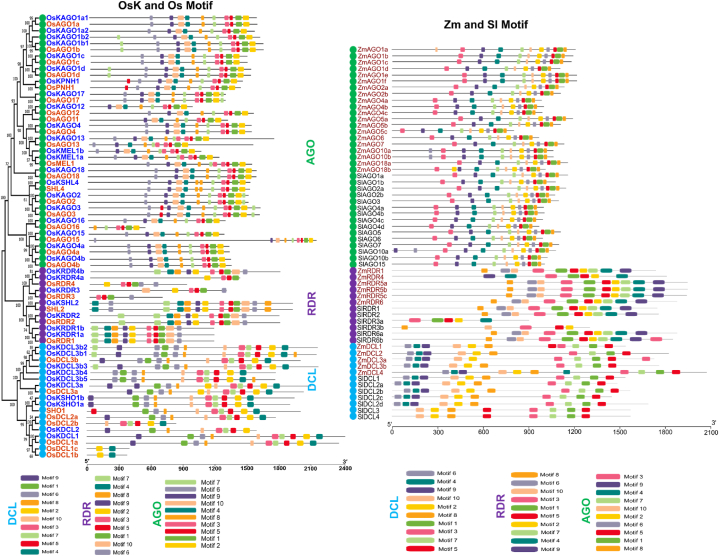
Motifs of OsKRNAi, OsRNAi, ZmRNAi, and SlRNAi proteins. Different colors represent different motifs.

Motifs are repetitive sequence patterns found in DNA and proteins that have a connection to particular functions. Using MEME-suite analysis, it has been selected ten significant motifs from OsKRNAi corresponding to OsRNAi, ZmRNAi, and SlRNAi proteins. The motif distributions of the majority of OsKDCL and OsDCL proteins have been observed to be almost identical. Similarly, OsKAGOs and OsAGOs have been shown similar conserved motifs except for OsKAGO17 (Fig. 3). In the RDR protein family, the OsKSHL2*,* OsKRDR1b*,* OsKRDR1a*,* OsKRDR2*,* OsKRDR4b*,* OsKRDR3*,* and OsKRDR4a have contained 9 out of 10 motifs of OsRNAi proteins with similar distributional patterns (Fig. 3).

#### 3.2.2 OsKRNAi genes structures analysis

**Fig. 4:**
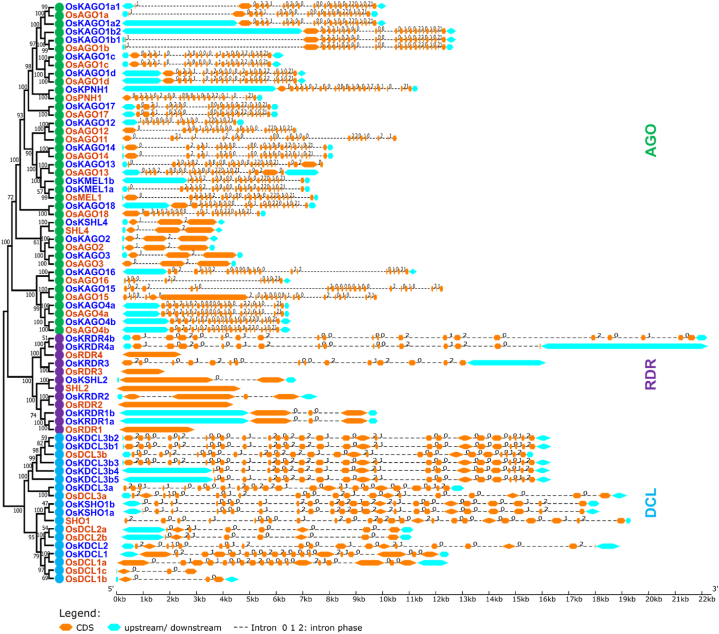
Structure of OsRNAi and OsKRNAi genes. The green color represents AGO, purple represents DCL, and paste represents RDR.

The exon-intron architecture of OsKRNAi has been studied in comparison to OsRNAi genes using the web tool GSDS. The gene structure of OsKDCLs has been revealed that they have 19–26 introns which are almost similar to OsDCLs (Fig. 4). The majority of OsKAGO genes exhibit a structural composition consisting of 20–23 introns. Only two introns have been found in *OsKAGO2, OsKAGO3,* and *OsKSHL4* genes. The gene structure of OsKDCLs revealed that they have 15–20 introns.

#### 3.2.3 Synteny analysis of OsKRNAi genes

**Fig. 5:**
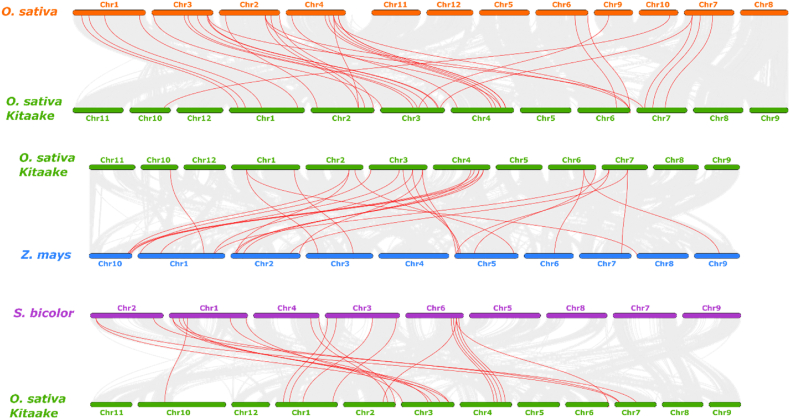
Synteny study of RNAi genes between *Kitaake* rice and other species. The grey lines show collinear blocks within the genome, and the red lines emphasize syntenic pairings of RNAi genes. The number of chromosomes is shown in the middle of each chromosome. Orange color represents rice, green color represents *Kitaake* rice, blue color represents maize, and purple color represents sorghum.

The investigation has focused on examining the phylogenetic link, probable evolutionary relationship, and member's collinearity of the RNAi gene family between *Kitaake* rice and other species, such as rice (*O. sativa*), maize (*Zea mays*), and sorghum (*Sorghum bicolor*). The collinearity relationship between RNAi genes across several species is displayed in Fig. 5. The OsKRNAi genes have different levels of collinearity with other species. The highest number of gene homologous pairs (33) among *O. sativa*, and *O. sativa Kitaake* and have a more recent evolutionary relationship. *Kitaake* rice has orthologous RNAi pairings, following by rice, maize, and sorghum, which have 33, 27, and 22 homologous pairs, respectively. This analysis suggests that *Kitaake* rice exhibits a stronger association with cereal plants (Fig. 5).

The calculation of Ka/Ks ratios for the OsKRNAi gene pairs has been conducted to investigate the comprehensive evolutionary understanding linked to the RNAi gene family. The results have shown that the Ka/Ks values of five duplicated OsKRNAi gene pairs have been less than one, indicating that these OsKRNAi genes have been subjected to purifying selective stress during their evolution (Table 2). The duplicate gene pairs exhibited divergence times ranging from 0.13 to 40.60 million years ago (Mya), with an average of 17.27 Mya.

**Table 2:** Ka/Ks value and divergent time of the duplicated OsKRNAi gene pairs.

Duplicated gene pairs	Ka	Ks	Ka/Ks	Divergence Time (MYA)
***OsKSHO1b/OsKSHO1a***	0.00514	0.007658	0.67110138	0.255278
***OsKAGO1c/OsKAGO1d***	0.12081	1.037608	0.116430965	34.58695
***OsKAGO4a/OsKAGO4b***	0.121078	1.218133	0.099396744	40.60442
***OsKAGO2/OsKAGO3***	0.227597	0.388332	0.586086974	12.94442
***OsKRDR4b/OsKRDR4a***	0.002208	0.003912	0.564283674	0.130412

#### 3.2.4 Sub-cellular localizations of OsKRNAi proteins

**Fig. 6:**
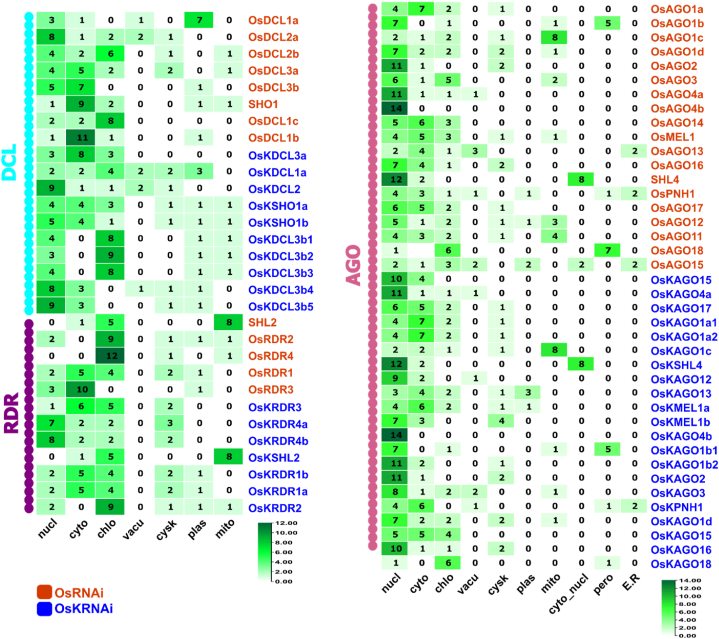
Sub-cellular localization analysis of OsRNAi and OsKRNAi proteins. The legend values indicate the number proteins that are closest to the query proteins (OsRNAi/OsKRNAi) in different sub-cellular locations. This analysis includes nucl: nucleus, cyto: cytosol, chlo: chloroplast, vacu: vacuolar membrane, cysk: Cytoskeletal, plas: plasma membrane, mito: mitochondrion, pero: peroxisome, E.R.: Endoplasmic Reticulum.

The investigation of sub-cellular localization has been conducted to gain insights into the closest neighbor proteins. The legend values provide information regarding the proximity of proteins to the query proteins (OsRNAi/OsKRNAi) across various sub-cellular locations. According to the sub-cellular localization analysis, most OsKAGO, OsKRDR, and OsKDCL proteins have nearest neighbors in the nucleus, chloroplast, and cytosol (Fig. 6). Several OsKRNAi proteins have neighbors in close proximity to cell membranes, plastids, and mitochondria (Fig. 6).

### 3.3 Gene ontology (GO) analysis of OsRNAi proteins

The GO enrichment study has been used to determine cellular constituents, molecular roles, and biological processes that are compared with OsKRNAi proteins and OsRNAi proteins. There is currently any GO information available for OsKRNAi genes. *Kitaake* is a variety of *Japonica* rice and as a result, this GO analysis has been performed using the genome of the *O. sativa* subsp. *Japonica*. This analysis has discovered annotated GO-terms of *O. sativa* subsp. *Japonica* that are abundant in both OsRNAi and OsKRNAi proteins. We independently verified the results for both OsRNAi and OsKRNAi using DeepGOWeb and obtained consistent findings. Therefore, we have not included the results in the article.

The various GO terms associated with the OsRNAi proteins explain the different biological pathways. In this study, 22 of 23 {OsAGO1a (OsKAGO1a1), OsAGO1b (OsKAGO1b1), OsAGO1c (OsKAGO1c), OsAGO1d (OsKAGO1d), OsAGO2 (OsKAGO2), OsAGO3 (OsKAGO3), OsAGO14 (OsKAGO14), OsAGO4a (OsKAGO4a), OsAGO4b (OsKAGO4b), OsMEL1 (OsKMEL1a), SHL2 (OsKSHL2), PNH1 (OsKPNH1a), OsAGO12 (OsKAGO12), OsAGO17 (OsKAGO17), OsAGO18 (OsKAGO18), OsDCL1a (OsKDCL1a), OsDCL2 (OsKDCL2), OsDCL3b (OsKDCL3b3), SHO1 (OsKSHO1a), OsRDR1a (OsKRDR1a), OsRDR2 (OsKRDR2), SHL4 (OsKSHL4a)} proteins have been involved in the process of gene silencing by RNA (GO:0031047), gene silencing (GO:0016458), negative regulation of gene expression (GO:0010629), negative regulation of macromolecule metabolic process (GO:0010605), negative regulation of metabolic process (GO:0009892), and negative regulation of biological process (GO:0048519) as biological processes. Three OsDCLs (OsKDCLs) {OsDCL1a (OsKDCL1a), OsDCL2 (OsKDCL2), SHO1 (OsKSHO1a)} proteins have been identified as being associated with RISC complex (GO:0016442) and RNAi effector complex (GO:0031332) as cellular components. There are 12 OsRNAi (OsKRNAi) {OsAGO1a (OsKAGO1a1), OsAGO2 (OsKAGO2), OsAGO4b (OsKAGO4b), OsMEL1 (OsKMEL1a), SHL2 (OsKSHL2), OsDCL1a (OsKDCL1a), OsDCL2 (OsKDCL2), OsDCL3b (OsKDCL3b3), SHO1 (OsKSHO1a), OsRDR1a (OsKRDR1a), OsRDR2 (OsKRDR2)} proteins have been identified as being associated with RNA binding (GO:0003723), 21 OsRNAi (OsKRNAi) {OsAGO1a (OsKAGO1a1), OsAGO1b (OsKAGO1b1), OsAGO1c (OsKAGO1c), OsAGO1d (OsKAGO1d), OsAGO2 (OsKAGO2), OsAGO3 (OsKAGO3), OsAGO14 (OsKAGO14), OsAGO4a (OsKAGO4a), OsAGO4b (OsKAGO4b), OsMEL1 (OsKMEL1a), PNH1 (OsKPNH1a), OsAGO12 (OsKAGO12), OsAGO17 (OsKAGO17), OsAGO18 (OsKAGO18), OsDCL1a (OsKDCL1a), OsDCL2 (OsKDCL2), OsDCL3a (OsKDCL3a), SHO1 (OsKSHO1a), OsRDR1a (OsKRDR1a), OsRDR2 (OsKRDR2), SHL4 (OsKSHL4a)} proteins have been identified as being associated with protein binding (GO:0005515) (Table 3) as molecular function. The OsAGOs (OsKAGOs) possess diverse biological activities that can be elucidated by evaluating their expression in any biological study. Other OsRNAi (OsKRNAi) pathway proteins have been identified as being associated with double-stranded RNA-specific ribonuclease activity, nucleic acid binding, RNA polymerase activity, miRNA binding and heterocyclic compound binding activity.

**Table 3:** Top eight significantly enriched GO-terms of OsRNAi and OsKRNAi proteins. Here BP for biological processes, MF for molecular functions, and CC for cellular components.

GO.ID	Term	OsRNAi (OsKRNAi) proteins
GO:0031047	gene silencing by RNA (BP)	OsAGO1a (OsKAGO1a1), OsAGO1b (OsKAGO1b1), OsAGO1c (OsKAGO1c), OsAGO1d (OsKAGO1d), OsAGO2 (OsKAGO2), OsAGO3 (OsKAGO3), OsAGO14 (OsKAGO14), OsAGO4a (OsKAGO4a), OsAGO4b (OsKAGO4b), OsMEL1 (OsKMEL1a), SHL2 (OsKSHL2), PNH1 (OsKPNH1a), OsAGO12 (OsKAGO12), OsAGO17 (OsKAGO17), OsAGO18 (OsKAGO18), OsDCL1a (OsKDCL1a), OsDCL2 (OsKDCL2), OsDCL3b (OsKDCL3b3), SHO1 (OsKSHO1a), OsRDR1a (OsKRDR1a), OsRDR2 (OsKRDR2), SHL4 (OsKSHL4a).
GO:0016458	gene silencing (BP)	OsAGO1a (OsKAGO1a1), OsAGO1b (OsKAGO1b1), OsAGO1c (OsKAGO1c), OsAGO1d (OsKAGO1d), OsAGO2 (OsKAGO2), OsAGO3 (OsKAGO3), OsAGO14 (OsKAGO14), OsAGO4a (OsKAGO4a), OsAGO4b (OsKAGO4b), OsMEL1 (OsKMEL1a), SHL2 (OsKSHL2), PNH1 (OsKPNH1a), OsAGO12 (OsKAGO12), OsAGO17 (OsKAGO17), OsAGO18 (OsKAGO18), OsDCL1a (OsKDCL1a), OsDCL2 (OsKDCL2), OsDCL3b (OsKDCL3b3), SHO1 (OsKSHO1a), OsRDR1a (OsKRDR1a), OsRDR2 (OsKRDR2), SHL4 (OsKSHL4a).
GO:0010629	negative regulation of gene expression (BP)	OsAGO1a (OsKAGO1a1), OsAGO1b (OsKAGO1b1), OsAGO1c (OsKAGO1c), OsAGO1d (OsKAGO1d), OsAGO2 (OsKAGO2), OsAGO3 (OsKAGO3), OsAGO14 (OsKAGO14), OsAGO4a (OsKAGO4a), OsAGO4b (OsKAGO4b), OsMEL1 (OsKMEL1a), SHL2 (OsKSHL2), PNH1 (OsKPNH1a), OsAGO12 (OsKAGO12), OsAGO17 (OsKAGO17), OsAGO18 (OsKAGO18), OsDCL1a (OsKDCL1a), OsDCL2 (OsKDCL2), OsDCL3b (OsKDCL3b3), SHO1 (OsKSHO1a), OsRDR1a (OsKRDR1a), OsRDR2 (OsKRDR2), SHL4 (OsKSHL4a).
GO:0003723	RNA binding (MF)	OsAGO1a (OsKAGO1a1), OsAGO2 (OsKAGO2), OsAGO4b (OsKAGO4b), OsMEL1 (OsKMEL1a), SHL2 (OsKSHL2), OsDCL1a (OsKDCL1a), OsDCL2 (OsKDCL2), OsDCL3b (OsKDCL3b3), SHO1 (OsKSHO1a), OsRDR1a (OsKRDR1a), OsRDR2 (OsKRDR2).
GO:0005515	protein binding (MF)	OsAGO1a (OsKAGO1a1), OsAGO1b (OsKAGO1b1), OsAGO1c (OsKAGO1c), OsAGO1d (OsKAGO1d), OsAGO2 (OsKAGO2), OsAGO3 (OsKAGO3), OsAGO14 (OsKAGO14), OsAGO4a (OsKAGO4a), OsAGO4b (OsKAGO4b), OsMEL1 (OsKMEL1a), PNH1 (OsKPNH1a), OsAGO12 (OsKAGO12), OsAGO17 (OsKAGO17), OsAGO18 (OsKAGO18), OsDCL1a (OsKDCL1a), OsDCL2 (OsKDCL2), OsDCL3a (OsKDCL3a), SHO1 (OsKSHO1a), OsRDR1a (OsKRDR1a), OsRDR2 (OsKRDR2), SHL4 (OsKSHL4a).
GO:0004525	ribonuclease III activity, (MF)	OsDCL1a (OsKDCL1a), OsDCL2 (OsKDCL2), OsDCL3a (OsKDCL3a), OsDCL3b (OsKDCL3b3), SHO1 (OsKSHO1a).
GO:0031332	RNAi effector complex, (CC)	OsDCL1a (OsKDCL1a), OsDCL2 (OsKDCL2), SHO1 (OsKSHO1a).
GO:0016442	RISC complex (CC)	OsDCL1a (OsKDCL1a), OsDCL2 (OsKDCL2), SHO1 (OsKSHO1a).

### 3.4 OsKRNAi gene regulatory network analysis

Gene expression regulation is controlled by gene regulatory elements through biochemical interactions involving DNA, chromatin, and TFs. No information regarding TFs, *cis*, and miRNA is presently accessible for OsKRNAi genes. *Kitaake* is a variety of *O. sativa*, and as a result, this regulatory element analysis was performed using the genome of the *O. sativa*. This study has found that TFs, *cis*, and miRNAs of *O. sativa* subsp. *Japonica* is present in OsKRNAi.

#### 3.4.1 *Trans*-regulatory factors (TFs) of OsKRNAi genes

**Fig. 7:**
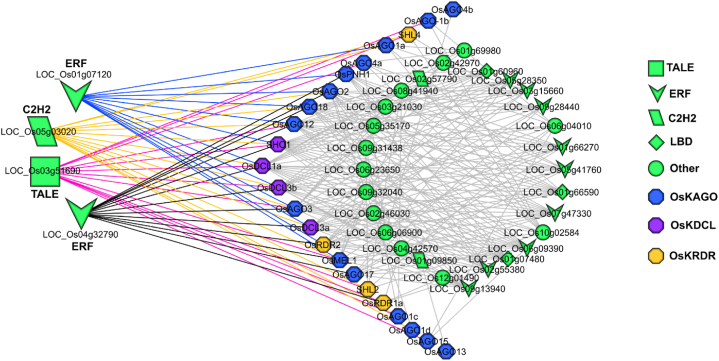
The regulatory network among the TFs and OsKRNAi genes. The network nodes are colored based on RNAi genes (AGOs purple, DCLs blue, RDRs orange) and TFs (green).

The TFs study has been investigated how transcription factors influenced OsRNAi (OsKRNAi) gene expression. In this scenario, identify transcription factors that have been annotated for OsRNAi genes but have been predicted for OsKRNAi genes. This work has identified a total of 32 TFs that have significant associations with OsKRNAi genes (Fig. 7). The TF families have identified that TFs can be classified into seven significant types. The top four ERF, LBD, TALE, and C2H2 families have included 113, 30, 20, and 18 TFs, representing 80 % of all detected TFs. TALE, TCP, NAC, and MYB have been related to the four more significant TFs uncovered in this investigation. The ERF (Ethylene Response Factor) family of TFs has a close connection with some OsRNAi (*OsAGO1a, OsAGO2, OsAGO3, OsAGO4a, OsMEL1, OsPNH1, OsAGO11, OsAGO12, OsAGO15, OsAGO17, OsAGO18, OsDCL1a, OsDCL1c, OsDCL3a, OsDCL3b, SHO1, OsRDR1 and OsRDR2*) and OsKRNAi genes, including *OsKAGO-1a1, OsKAGO-2, OsKAGO-3, OsKAGO-4a, OsKMEL1a, OsKPNH1a, OsKAGO-12, OsKAGO-17, OsKAGO-18, OsKDCL-1a, OsKDCL-3a, OsKDCL-3b3, OsKSHO1a, OsKRDR-1a,* and *OsKRDR-2* (Fig. 7). LBD (Lateral Organ Boundaries Domain) transcription factors are a protein family that is unique to plants and plays important roles in many areas of their development. The LBD family of TFs has a close connection with some OsRNAi (*OsAGO1a, OsAGO3, OsAGO4, OsMEL2, OsPNH1, OsPNH2, OsAGO11, OsAGO13, OsAGO15, OsAGO19, OsDCL1a, OsDCL1c, OsDCL3a, OsDCL3b, SHO1, SHO1,* and *OsRDR3*) and OsKRNAi genes, including *OsKAGO1a1, OsKAGO2, OsKAGO3, OsKMEL1a, OsKPNH1a, OsKAGO12, OsKAGO18, OsKDCL1a, OsKDCL3a, OsKDCL3b3, OsKSHO1a* and *OsKRDR2.* TALE (Three-amino-acid-loop-extension) transcription factors are a broad and diverse class of proteins discovered in plants. The TALE family of TFs has a close connection with some OsRNAi (*OsAGO1b, OsAGO1c, OsAGO3, OsAGO4, OsMEL1, OsAGO11, OsAGO12, OsDCL1a, OsDCL1b, OsDCL3b, SHO1, OsRDR1, SHL2* and *SHL4*) and OsKRNAi genes, including *OsKAGO3, OsKMEL1a, OsKDCL1a, OsKDCL3b3, OsKSHO1a, OsKRDR1a* and *OsKRDR4b.* The C2H2 family of TFs has a close connection with some OsRNAi (*SHL2*) and OsKRNAi genes, including *OsKAGO4a* and *OsKRDR4b*. In addition, the node degree analysis has identified four hub TFs that have more than five interaction partners with RNAi genes. This discovery suggests that certain TFs may play a role in RNAi gene regulation.

#### 3.4.2 Cis-regulatory factors of OsKRNAi genes

**Fig. 8:**
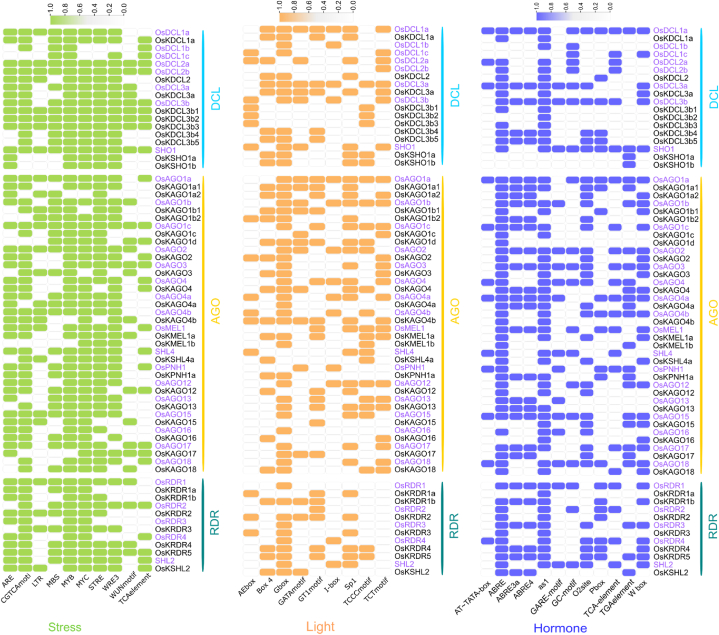
The *cis*-acting regulatory network analysis of OsRNAi (Purple color) and OsKRNAi (Black color) genes. The values 0 and 1 denote the absence and presence of that element in the corresponding genes.

The present study has examined the role of *cis*-acting regulatory elements (CAREs) in regulating the expression of OsKRNAi genes in comparison to OsRNAi genes. This result indicated the presence of stress-responsive (SR), light-responsive (LR), hormone-responsive (HR), and other activity-related motifs in the regulatory regions of the OsKRNAi and OsRNAi genes (Fig. 8). According to this study, the OsKRNAi and OsRNAi genes are associated with significant SR motifs such as the ARE, CGTCA-motif, MYB, MYC, STRE, TCA-element, and WRE3. TCA-element is mostly found in OsRNAi but not in OsKRNAi genes. WUN-motif is mostly found in OsKRNAi but not in OsRNAi genes. The OsKRNAi and OsRNAi genes highly shared LR motifs, AE-box, Box 4, G-box, and Sp1. TCT-motif is absent in RDR genes in both varieties. I-Box is mostly found in OsRNAi but not in OsKRNAi genes. GATA-motif is mostly found in OsKRNAi but not in OsRNAi genes. According to this study, the OsKRNAi and OsRNAi genes possess significant amounts HR motifs, such as the ABRE, ABRE3a, ABRE4, as-1, O2-site, TGA-element, and W box. At-TATA-box, TCA-element and GC-motif are mostly found in OsRNAi but not in OsKRNAi genes.

#### 3.4.3 miRNA of OsKRNAi genes

**Fig. 9:**
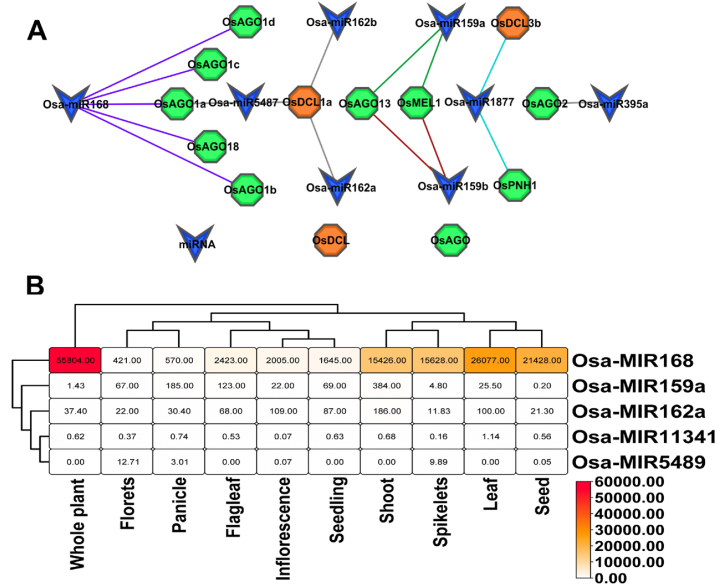
(a) The regulatory network among the miRNA and OsRNAi (OsKRNAi) proteins. (b) miRNA expression level in different plant organs. DCL and AGO proteins were represented in orange and green, respectively, and miRNAs were presented in blue. with 100 μM rapamycin.

The present study has examined the regulatory role of micro-RNA (miRNA) on the expression of OsRNAi (OsKRNAi) genes. In this case, miRNAs are already annotated for OsRNAi genes but predicted for OsKRNAi genes. This study has found 8 important miRNAs, Osa-miR395 and Osa-miR5487, using *O. sativa* genome. Osa-miR168 interacts with 7 RNAi genes: *OsAGO18* (*OsKAGO18*), *OsAGO1a* (*OsKAGO1a*), *OsAGO1b* (*OsKAGO1b*), *OsKAGO1c* (*OsAGO1c*), and *OsKAGO1d* (*OsAGO1d*) (Fig. 9(a)). Osa-miR168 is highly expressed throughout the whole rice plant (Fig. 9(b)).

### 3.5 Gene expression of OsKRNAi genes

**Fig. 10:**
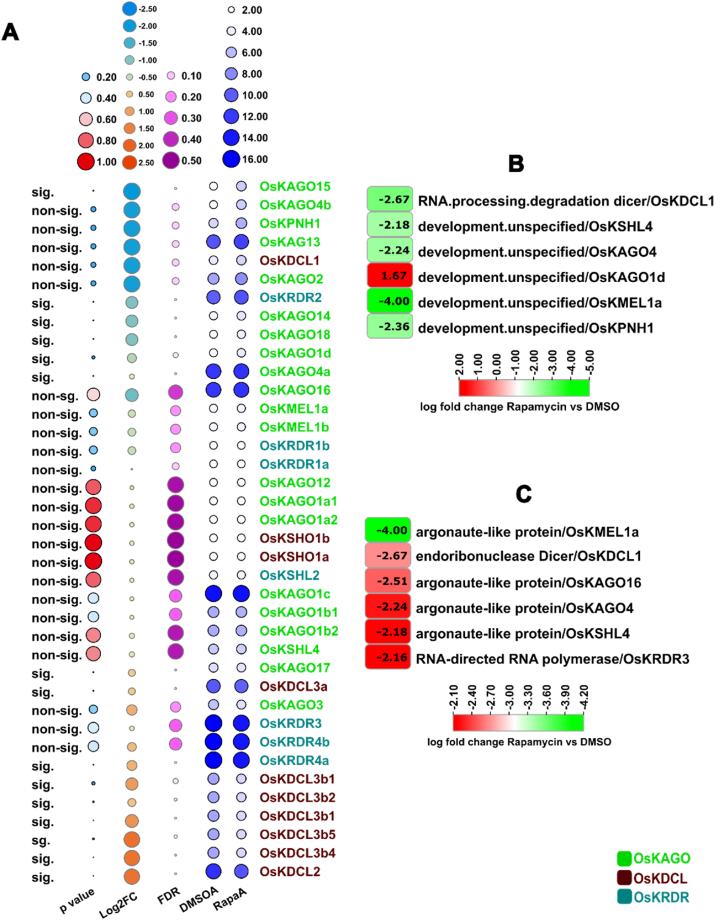
(a) Analysis of the OsKRNAi expression level using various statistical methods. sig: significant, non-sig: Non-significant, DMSOA: dimethyl sulfoxide average, RapaA: rapamycin average, the significant difference at p < 0.05. **(b) OsKRNAi expression in different biological processes. (c) OsKRNAi expression in different annotations.**

An *in-silico* investigation of gene expression was conducted to determine the biological presence of the OsKRNAi gene in rice. Analysis of the expression data in different locus expression of *Kitaake* rice cells have treated with the TOR-specific inhibitor rapamycin. This study has been based on the NCBI GEO database, dataset (GSE93872) for BioProject ID: PRJNA362628 locus ID expressions data of *Kitaake* rice. The study has reported a substantial expression of *OsKAGO15* (LOC_Os04g06770)*, OsKRDR2* (LOC_Os04g39160)*, OsKAGO4* (LOC_Os07g09020)*, OsKAGO18* (LOC_Os07g28850)*, OsKAGO1d* (LOC_Os06g51310)*, OsKAGO4a* (LOC_Os01g16870)*, OsKAGO17* (LOC_Os02g07310)*, OsKDCL3a* (LOC_Os01g68120)*, OsKRDR4a* (LOC_Os01g10140)*, OsKDCL3b1* (LOC_Os10g34430)*, OsKDCL3b2* (LOC_Os10g34430)*, OsKDCL3b1* (LOC_Os10g34430)*, OsKDCL3b5* (LOC_Os10g34430)*, OsKDCL3b4* (LOC_Os10g34430) and *OsKDCL2* (LOC_Os03g38740) (Fig. 10 (a)). The expression of the genes *OsKDCL1* (LOC_Os03g02970), *OsKSHL4* (LOC_Os03g33650), *OsKAGO4* (LOC_Os07g09020), *OsKMEL1a* (LOC_Os03g58600), and *OsKPNH1* (LOC_Os06g39640) is downregulated, but *OsKAGO1d* (LOC_Os 06g51310), overexpression of this gene is observed in many biological processes, such as RNA processing degradation and unspecified development when *Kitaake* rice cells are treated with 100 μM rapamycin for 24 h (Fig. 10 (b)). The *OsKMEL1a* (LOC_Os03g58600), *OsKDCL1* (LOC_Os03g02970), *OsKAGO16* (LOC_Os01g16870), *OsKAGO4* (LOC_Os07g09020), *OsKSHL4* (LOC_Os03g33650), and *OsKRDR3* (LOC_Os01g10140) genes undergo downregulation in argonaute-like protein, endoribonuclease dicer, RNA-directed RNA polymerase annotations, when *Kitaake* rice cells are treated for 24 h (Fig. 10 (c)).

## 4 Discussion

RNA interference (RNAi) genes assist plants in protecting themselves against both exogenous parasites and endogenous pathogens, regulating the expression of genes that encode proteins. DCL proteins convert dsRNA from viruses or parasites into siRNAs, which are then incorporated into RISC, which directs AGO proteins to target and destroy viral or parasitic RNA [95,96]. The key RNAi genes—RDR, DCL, and AGO—play vital roles in regulating various biological processes and pathways [97,98]. In this analysis, we have identified 10 OsKDCLs, 21 OsKAGOs, and 7 OsKRDRs as RNAi proteins from the *Kitaake* rice genome by BLASTp search govern by the 8 OsDCLs, 19 OsAGOs, and 5 OsRDRs of *Oryza sativa* (OsRNAi) proteins as parental species, highlighting their locations, regulators, biological processes, domains, motifs, structures, and molecular roles [54].

The predicted RNAi protein groups {OsKSHO1a, OsKSHO1b}, {OsKAGO1 (a-d)} and OsKSHL2 may enhance the early phases of floral/panicle development since they belong to the gene groups {SHO1}, {OsAGO1 (a-d)} and {SHL2}*,* respectively. Due to its similarity to OsPNH1, the OsKPNH1 protein may have the potential to influence shoot apical meristem (SAM) and leaf development. The OsKRDR2 protein may influence flower development since it is similar to OsRDR2 (Fig. 1) [54]. The downregulation of OsDCL4 (OsKSHO1a, OsKSHO1b) protein makes it easier for viruses to infect rice [99]. OsDCL2 (OsKDCL2) is highly expressed in rice egg cells, and DCL2 increases the egg cell's tolerance to viruses and other external stressors [100]. The OsDCL4, OsDCL3b (OsKDCL3b1 to b5), and OsDCL1 (OsKDCL1) proteins are components of the rice 21- and 24-nucleotide phased sRNA biosynthesis pathway in rice [[101], [102], [103]]. The OsAGO1 (OsKAGO1a to OsKAGO1d), OsAGO1b (OsKAGO1b1, OsKAGO1b2) proteins play a crucial role in rice growth and development [104]. The OsAGOs (OsKAGOs) protein is crucial in siRNA and miRNA pathways, hormone response, and regulating rice's vegetative and reproductive development [105]. A shortage of OsAGO18 (OsKAGO18) heightens vulnerability, while its excessive expression enhances viral resilience [106,107]. The OsAGO17 (OsKAGO17) is a protein that positively affects grain size, pollen, and weight in rice and may be a key element in the sRNA pathway [[108], [109], [110]]. The OsPNH1 (OsKPNH1) is involved in leaf development [111]. Overexpression of OsAGO2 (OsKAGO2) protein results in an improvement of salt tolerance as well as grain length [112,113]. Genes (such as *OsKAGO1d*) have been found to be overexpressed in target of rapamycin (TOR), increasing susceptibility to pathogens [114]. Rice's tolerance to rice stripe virus (RSV) infection is improved by lower OsRDR1 (OsKRDR1a*,* OsKRDR1b) expression [115] and OsRDR6 (OsKSHL2) protects rice plants against double-stranded RNA virus [116]. Antisense OsRDR6AS reduced OsRDR6 expression by 70–80 % [117], but RSV infection and signs were significantly raised via the RNAi-mediated antiviral immunity pathway [118]. RNAi-based antiviral defense is a mechanism in plants that uses tiny RNA molecules to suppress viruses [119]. Salicylic acid, a plant hormone, activates plant-encoded RDR, which then increases antiviral RNAi in infected tissues. This stops the spread of the virus by giving stem cells information about the RNA-based virus sequence [120]. The initial antiviral defense mechanism is initiated by viral siRNAs direct RNAi to specifically target viral RNA and DNA within infected plant cells [121].

More specifically, the PIWI domain that has been found may regulate the expression of mRNA [122]. Rice DEAD-box RNA helicases [123] and wheat DEAD-box RNA helicases (TaDEAD-box) [124] are similar to identified OsKDEAD-Box may improve resilience to drought, salt, and cold stress in *Kitaake* rice (Fig. 2). OsKDCLs, like other DCLs, have multiple exons and may play a role in chromatin remodeling and DNA degradation [125,126] (Fig. 4). In this study, the number of RNAi gene homologous pairs (33) is highest in *O. sativa* and *O. sativa Kitaake* while compared to *O. sativa, O. sativa Kitaake, Sorghum bicolor*, and *Zea mays,* indicating a higher degree of similarity and a closer evolutionary relationship. This is because *O. sativa Kitaake* is a cultivar of *O. sativa* (Fig. 5). The duplicate gene pairs exhibited divergence times ranging from 0.13 to 40.60 million years ago (Mya), with an average of 17.27 Mya (Table 2). DICER (OsKDCLs), ARGONAUTE, (OsKAGOs), and those are nuclear localized and plays a critical role in sRNA biogenesis and degrade/blocked mRNA of targeted gene (Fig. 6) [127,128].

The terms of the molecular function, such as ribonuclease III activity, have been related to five OsKDCL proteins [129] (Table 3) which precisely breaks down dsRNAs for RNAi mechanisms in plants [130,131]. The OsKDCL2 protein may play an active role in biotic stress responses by stimulating gene silencing by RNA through the molecular processes of ribonuclease III function and RNA binding [132,133]. Phylogenetic and collinearity analyses provide crucial information on the functional relationships between genes. Genes that are physically close are frequently involved in the same biological processes or pathways. OsRNAi, ZmRNAi, and SbRNAi assist in defending against both abiotic and biotic stresses [[134], [135], [136]]. Considering the phylogenetic and syntenic similarities, OsKRNAi may have evolved alongside these genes and may act in response to biotic and abiotic stresses (Fig. 5).

The enriched biological processes (GO-terms) with OsKRNAi genes including gene silencing by RNA [137] and gene silencing [138] have been annotated with OsRNAi genes. The OsKRNAi genes have been found to be linked with the RISC. RISC (RISC members: AGO, DICER, and others) is a multiprotein assembly that plays a crucial role in the RNAi pathway. It is composed of several proteins, including AGO proteins, which are essential for target RNA recognition and cleavage. Other components of RISC include Dicer, a protein that processes double-stranded RNA (dsRNA) into small RNA molecules, and various associated proteins [139]. The molecular function (GO-terms) RNA binding of the OsKRNAi genes have been annotated in rice [140] and protein binding [141].

Transcriptional regulation is fundamental to numerous physiological processes that plants undergo during their development, evolution, and adaptation to their surroundings [142]. The expression of RNAi genes (SiDCLs, SiAGOs, and *SiRDR07*) and ERF (SiERF) TF is increased during drought stress in foxtail millet, suggests a potential relationship due to their similar functions [143]. The OsNF-YB1 associated with ERF affects the grain and endosperm development of rice [144] and maize development [145] (Fig. 7). Overexpression of LBD TFs (*OsLBD37* and *OsLBD38*) resulted in a delayed maturation time and higher yield, consistent with the findings of predicted LBD TF [146]. TALEs play a significant role in improving wheat's resilience to drought and salinity stress [147]. ZmC2H2 gene expression is directly correlated with increased cold tolerance in maize [148]. Multiple genes that function downstream of LBD TF and regulate postembryonic root development are conserved in monocots and dicots. In addition, LBD gives evidence that particular genes are engaged in the process of shoot-derived roots in rice [149]. Maize ZmMYB3R TF, rice, and *Arabidopsis* MYB TF (identified MYB) are interconnected, showing their function in drought and salt stress tolerance [150] and developmental processes [151]. Identified NAC TF may possess vital and pivotal roles in the paper mulberries [152], rice [153], and wheat (TaNAC30) [154] salt, drought, water insufficiency, and cold stress response. Maize ZmTCP42 TF [155] and wheat TaTCP9 TF [156] (identified TCP) contribute to drought tolerance and enhanced spike and grain lengths. OsKRNAi genes have similar TFs that may have the same functions as OsRNAi genes. Considering the discussion above, since these crops belong to the Poaceae family, the TFs of OsKRNAi may play a similar role in *Kitaake* rice.

The identified SR-related motifs (ARE, MBS, TC-rich repeats, STRE, LTR, WRE3, and WUN-motif) have found in wheat that assist the plant surviving abiotic stress (Fig. 8) [157]. Because *Kitaake* and wheat are both members of the Poaceae family, those SR-motifs may serve similar functions in *Kitaake* rice. It has been found that the predicted LR-motifs, including AE-box [158], G-box [159], GATA-motif [160], GT1-motif [161], and Sp1 [162], play an important role in photosynthetic process of sorghum leaves [163]. Because *Kitaake* and sorghum are both members of the Poaceae family, those LR-motifs may serve similar functions in *Kitaake* rice. From above discussion *cis*-elements of OsKRNAi genes may play a vital role in enhancing *Kitaake* rice's stress tolerance.

MicroRNAs (miRNAs) play a crucial role in regulating eukaryotic gene expression by targeting messenger RNAs (mRNAs) for either breakage or translational inhibition [164]. The miRNAs that we have identified in OsKRNAi are also present in OsRNAi genes. The Osa-miR162, Osa-miR162a, Osa-miR168, Osa-miR159a, and Osa-miR159b as the post-transcriptional regulators of RNAi genes that improve the rice blast resistance and immunity to *M. oryzae* [[165], [166], [167]] (Fig. 9). We have found several crucial miRNAs (Osa-miR168, Osa-miR395, Osa-miR1877, Osa-miR5487) that can potentially assist in reducing the duration of flowering, promoting plant growth, and improving the resilience of *Kitaake* rice through OsKRNAi genes [[168], [169], [170], [171]].

The gene expression analysis has undertaken to ensure the existence of the OsKRNAi gene in *Kitaake* rice by using transcriptomic data. Plants with lower TOR levels are more resistant to bacterial and fungal infections than TOR overexpression lines. The *OsKAGO1d* gene is regulated in cultured *Kitaake* rice cells have been treated with less TOR by biological processes such as RNA processing degradation dicer. Many defense-related genes are suppressed by TOR overexpression [172] (Fig. 10). Genes (such as *OsKAGO1d*) were found to be overexpressed in target of rapamycin (TOR), increasing susceptibility to pathogens. Thus, the anticipated OsKRNAi genes may improve *Kitaake* rice growth and development by regulating the corresponding protein-coding genes in response to various biotic and abiotic challenges.

## 5 Conclusion

According to this study, the identified OsKRNAi genes are associated with stress response, protein-coding gene silencing, and resistance to disease. This study is an initial approach to identifying the key RNAi (DCLs, AGOs, RDRs) gene families in *Kitaake* rice. As a result, the insights gained from this work could be useful resources for improving *Kitaake* rice quality through the regulation of target-specific protein-coding genes to respond to both biotic and abiotic challenges.

## 6 Future studies

A wet-lab based experimental validation is required to confirm the performance of the identified OsKRNAi genes and associated components against different biotic and abiotic stresses.

## CRediT authorship contribution statement

**Md Darun Naim:** Writing – original draft, Formal analysis, Conceptualization. **Md Alamin:** Writing – review & editing. **Md Parvez Mosharof:** Writing – review & editing. **Ahmed Imtiaj:** Writing – review & editing, Conceptualization. **Md Nurul Haque Mollah:** Writing – review & editing, Supervision, Conceptualization.

## Data availability

The datasets that have been generated and analyzed during the current study are available:

For *Kitaake* rice, in the GenBank ID: GCA_009797565.1, under BioSample ID: SAMN08824567, BioProject ID: PRJNA448171, NCBI taxonomy ID: 39947, Phytozome genome ID: 499. Database link (https://phytozome-next.jgi.doe.gov/info/OsativaKitaake_v3_1):

For parent species *Oryza sativa*, in the GenBank ID: GCA_001433935.1, under BioSample ID: SAMD00000397, BioProject ID: PRJDB1747, NCBI taxonomy ID: 39947. Database link: (http://rice.uga.edu/).

## Funding

There was no specific funding for this work.

## Declaration of Competing Interest

The authors declare that they have no known competing financial interests or personal relationships that could have appeared to influence the work reported in this paper.

## Appendix A Supplementary data

Multimedia component 1 The GO-terms of *O. Sativa* enrichment analysis by RNAi gene-set. Multimedia component 1
